# Renal Involvement in IgG4-Related Disease: From Sunlight to Twilight

**DOI:** 10.3389/fmed.2021.635706

**Published:** 2021-03-31

**Authors:** Riccardo Capecchi, Domenico Giannese, Diego Moriconi, Angelo G. Bonadio, Federico Pratesi, Cristina Croia, Maria F. Egidi, Ilaria Puxeddu, Antonio G. Tavoni, Paola Migliorini

**Affiliations:** ^1^Clinical Immunology and Allergy Unit, Department of Clinical and Experimental Medicine, University of Pisa, Pisa, Italy; ^2^Nephrology, Dialysis and Transplantation Unit, Azienda Ospedaliero Universitaria Pisana, Pisa, Italy; ^3^Department of Clinical and Experimental Medicine, University of Pisa, Pisa, Italy; ^4^Department of Surgical, Medical and Molecular Pathology and Critical Care Medicine, University of Pisa, Pisa, Italy; ^5^Pathology Unit, University of Pisa, Pisa, Italy

**Keywords:** IgG4-related disease, tubulointerstitial nephritis (TIN), retroperitoneal fibrosis, membranous nephropathy, ANCA - associated vasculitis

## Abstract

IgG4-Related Disease (IgG4-RD) is a fibroinflammatory condition characterized by a typical histopathological pattern (dense lymphoplasmacytic infiltrate with prevalent IgG4+ plasma cells and storiform fibrosis), which may involve the kidney both directly (IgG4-related kidney disease, IgG4-RKD) or indirectly, as a consequence of post-renal ureteral obstruction due to retroperitoneal fibrosis (IgG4-RD RF). The most frequent presentation of IgG4-RKD is IgG4-related tubulointerstitial nephritis (TIN), but a glomerular disease can be present, in most of the cases a membranous nephropathy. Albeit steroid-responsive, in some cases renal manifestations may lead to progressive and permanent organ damage. In this review we describe four clinical cases representative of typical and less typical renal manifestations of IgG4-RD, emphasizing a potential, subclinical, early involvement of the kidney in the disease.

## Introduction

IgG4-RD is a rare fibroinflammatory disorder that can affect almost any organ, characterized by lymphoplasmocytoid infiltrate, obliterative phlebitis, and storiform fibrosis often associated with eosinophilia and increased levels of IgG4 (1). Rigorous epidemiological studies on IgG4-RD have not yet been conducted. The estimated frequency of autoimmune pancreatitis in Japan varies between 0.28 and 1.08 per 100,000 people with 336–1,300 new cases per year (2), but these data can underestimate the prevalence of the disease, especially when other organs are involved. Males are more frequently affected, with a peak of incidence around the age of 60.

The pathogenesis of IgG4-RD is not clear: according to a generally accepted view, a persistent antigenic stimulus, perhaps from chronic infection, induces an increased polyclonal expansion of B cells under the influence of M2 macrophages, CD4+ (mainly cytotoxic) T cells and Tfh, in a milieu of IL-4, IL-10, IL-1beta, and TGF-beta cytokines. These signals promote IgG4 class switching, somatic mutation, and plasma cells expansion, as well as a local fibrotic response (3).

Clinical manifestations are various and can affect almost any organ, with pancreas, retroperitoneum, lymph nodes, and salivary glands as the most frequently involved ones. Disease presentation is usually chronic, sometimes paucisymptomatic and insidious, without high inflammatory manifestations, and characterized mainly by the development of mass lesions that exhibit the same histological pattern in all the organs affected. The rich infiltrate of IgG4+ plasma cells and CD4+ T cells is embedded in extensive fibrosis that assume the typical, distinctive whirled arrangement called “storiform.” Obliterative phlebitis is another feature of the disease. Eosinophils are frequently detected, while necrosis is rare; in fact, the presence of necrosis or granuloma or giant cells should rather suggest a different diagnosis (4). In peripheral blood, IgG4 levels are usually elevated, as well as circulating plasmablasts. On the basis of these features, IgG4-RD diagnostic criteria were proposed (5), by which a patient can be stratified as “probable,” “possible,” or “definite” IgG4-RD if serum IgG4 levels above 135 mg/dL are (or not) associated to a number of IgG4+ plasma cells > 10/HPF and a IgG4+/IgG+ ratio >40% for most tissues in the context of a tumefactive lesion. Exceptions to this rule were proposed for some organ-specific manifestations, such as Mikulicz disease or Autoimmune Pancreatitis (AIP), for whom specific diagnostic criteria were already present, with the possibility for AIP to make a definite diagnosis without histological specimen if the pancreas presented suggestive radiological aspects in association with increased level of IgG4 (5).

Recently, ACR/EULAR classification criteria were proposed (6). Developed and validated in a large international cohort of patients, these criteria had an excellent performance in discriminating IgG4-RD from disease mimics. These classification criteria contain entry, exclusion, and inclusion criteria; these latter allow the diagnosis of IgG4-RD when a score of 20 points is reached. One of the exclusion criteria proposed by ACR/EULAR classification is the scarce response to steroid treatment. In fact, responsiveness to glucocorticoids is a main clinical characteristic of IgG4-RD, even if relapses are common after steroid discontinuation (3). DMARDs such as methotrexate, azathioprine, hydroxychloroquine, and tacrolimus are used, but their efficacy is reported in limited studies. Mycophenolate and cyclophosphamide were evaluated in two trials and both drugs reduced, but not eliminated, relapses. Rituximab is an anti CD20 monoclonal treatment that demonstrates a dramatic efficacy in IgG4-RD, thanks to depletion of B cells and reduction of inflammatory infiltrate: treatment with Rituximab in the early stages of the disease can reverse fibrosis. Even in this case, clinical remission may last 6–18 months. Recently, a long-term efficacy of rituximab was evaluated when used every 6 months for maintenance therapy, an approach already used in ANCA-associated vasculitis (7).

As soon as the multisystem involvement of IgG4-RD became clear, it was appreciated that kidney was a prominent target organ. Reported frequency of renal involvement is in fact comprised between 10 and 27% of the cases (8, 9).

We report 4 cases of IgG4-RD with renal involvement that exemplify the different pattern of renal disease that can be observed in this disorder, outlining the clinical course and therapeutic approach.

Informed consent was obtained from each patient, for every procedure performed.

## Case Presentations

### Case 1

A 39 years old clergyman with a history of asthma, arterial hypertension and gastroesophageal reflux came to our attention for exophthalmos, previously treated with steroid boluses, and submandibular tumefactive lesions. His right parotid had echographic signs of parenchymal subversion and a Positron emission tomography/computed tomography (PET/CT), performed 3 years before admission, showed enlargement and high uptake of multiple lymph nodes in neck, mediastinus, tracheal, periaortic, and interaortocaval regions (SUVmax 7.3). To exclude hematologic malignancy, a lymph node needle biopsy was performed and showed hyperplasia with reactive plasmocytosis (IgG4 staining not performed). Blood tests showed hypergammaglobulinemia (19.6%), rheumatoid factor positivity (72.7 <30) and hypocomplementemia (C3 76 mg/dL, C4 6 mg/dL). Creatinine levels progressively increased from 1.19 to 1.92 mg/dL. At hospital admission the patient presented left eye exophthalmos and swelling of neck lymph nodes in the right side. Serum IgG4 were 1,300 mg/dL (49-66), IgG4/IgG ratio was 44.45% and circulating plasmablasts (CD19+ CD20– CD27+ CD38^bright^ cells) were highly increased (5,520 cells/mL; normal <635 cells/mL). An abdominal echography showed a hypoechogenic aspect of renal cortex. Urinary Retinal Binding Protein (RBP) and beta2microglobulin were increased (0.3 mg/dL with normal <0.1 and 337 mcg/L with normal <154 mcg/L, respectively), urinary light chain were present and urinary electrophoresis showed a mixed proteinuria (glomerular and tubular). Daily proteinuria was above 700 mg/24 h. Urinary sodium excretion, as well as potassium, calcium, and phosporous, were slightly reduced.

**Figure 1:**
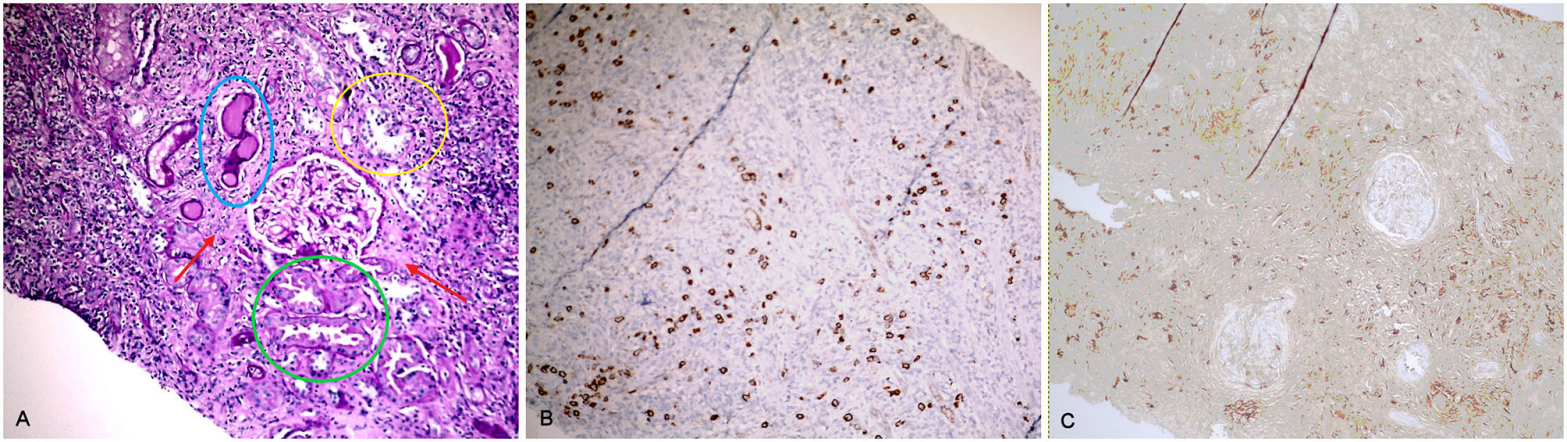
Histopathology of renal biopsy (case 1), showing IgG4-RD TIN. **(A)**: Diffuse tubulo-interstitial infiltrate associated with various degrees of tubular damage: initial thinning of tubular cells with cytoplasmic material in the tubular lumen (green circle); marked de-epithelialization of the tubular epithelium with inflammatory cells and tubulitis (yellow circle); tubular thyroidization due to disappearance of epithelial cells (blue circle); interstitial fibrosis is also evident (red arrow) (20x). **(B)**: CD138 staining, showing plasma cells in the inflammatory infiltrate (20x). **(C)**: immunohistochemistry for IgG4, showing positivity in the interstitium (20x).

A renal biopsy was performed. Examination of renal parenchyma showed a diffuse and intense fibrosis, with abundant lymphomonocytic and IgG4+ plasmacellular infiltrates (IgG4+/IgG+ ratio >40%). Immunohistochemistry for IgG4 demonstrated 22 IgG4 ± plasmacells/HPF. Only 2 out of 9 glomeruli were sclerotic, while tubuli were diffusely reduced and atrophic. Small and medium vessels showed mild thickening. No complement deposits were detected by immunofluorescence (Figure 1).

Histopathology of a neck lymph node had similar histological characteristics, with fibrotic areas enriched in plasmocytes (IgG4+/IgG+ ratio > 40%). The diagnosis of IgG4-Related Disease was made and treatment with monoclonal anti CD20 was started, with a progressive reduction of lymph node swelling and exophthalmos and a mild creatinine reduction, up to 1.4 mg/dL in 3 months.

### Case 2

**Figure 2:**
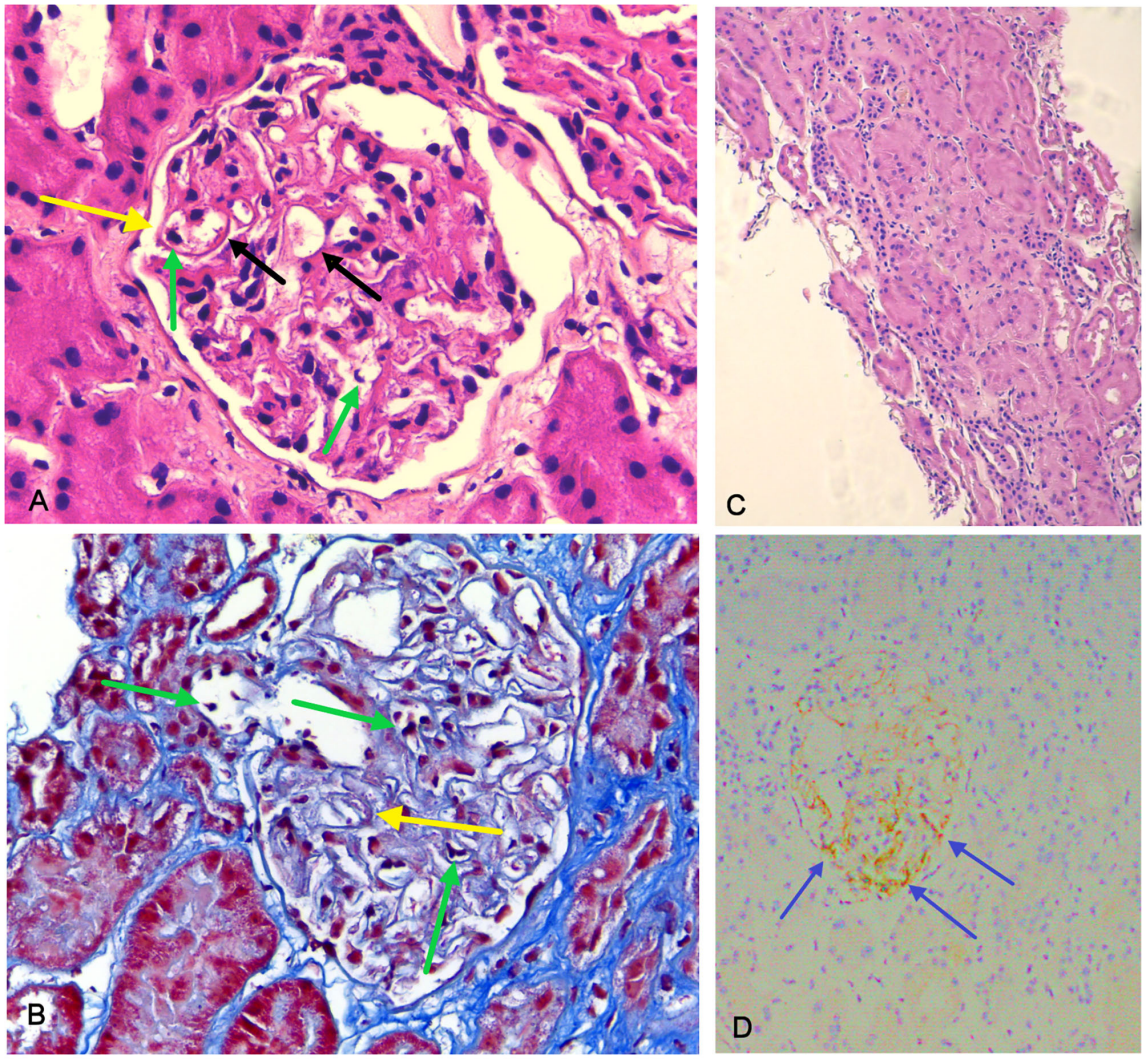
Histopathology of renal biopsy in case 2, indicative of membranous glomerulonephritis. Membrane (yellow arrow) and capillary loops (black arrow) thickening demonstrated by hematoxylin and eosin stain (**A**, 40x) and by Masson's trichrome stain (**B**, 10x). Some inflammatory cells (granulocytes) are present in the capillary lumens (green arrow), confirming the hypothesis of a secondary membranous glomerulonephritis. The interstitium, lacking infiltrates or fibrosis, and the tubules, arranged in a palisade, are not affected by the disease (**C**, 40x). IgG4 immunohistochemistry demonstrates positivity in the glomerular basal membrane (blue arrow) (**D**, 20x).

A 47 years old woman was evaluated in our outpatient clinics for AIP diagnosed 3 years before in another hospital (abdominal pain, hyperamilasemia; “Salt and pepper,” non-homogeneous pancreatic parenchyma at echography; serum IgG4 256 mg/dL). She had been treated with budesonide 9 mg/die and was under treatment with anti-IL-5 (Mepolizumab) for severe asthma. Blood tests showed increased ESR (108 mm/h, n.v. <25), fibrinogen (623 mg/dL, n.v. 200–450) and pancreatic amylase 150 U/L (n.v. 15–53); urinalysis showed microalbuminuria and increased number of white and red cells in the sediment. Urinary electrophoresis showed mixed proteinuria, mainly tubular. A PET/CT scan detected only low uptake on aortic arc, while abdominal RM did not show parenchymal involvement. Anti IL-5 therapy was stopped. Slowly, within 4 months, she developed lower limbs edema, and a rapid decay of her health status. At hospital admission she had proteinuria in the nephrotic range (6 g/L), increased ESR and fibrinogen, hyperamylasemia, ANA 1:160 speckled, normal creatinine level, normal C3 and C4, total IgG 301 with IgG4 81 mg/dL. Anti PLA2R and ANCA were negative and urinary electrophoresis showed mixed proteinuria. An echography showed globular aspect of pancreas, hyperechogenicity of renal cortical and medullary columns, linear hyperechogenic bands in salivary glands. A renal biopsy was performed, showing mild thickening of glomerular basal membrane and focal mesangial expansion; minimal interstitial fibrosis and IgG4-negative lymphomonocytic infiltrate; minimal tubular atrophy (5% of tubuli) in the absence of vascular abnormalities. IgG4 staining showed focal IgG4 on glomerular basal membrane, while by immunofluorescence deposits of IgG and C3 were detected on basal membrane (Figure 2), with very few IgG1 positive cells but no IgG2 or IgG3 positive cells (see Supplementary Figure 1). Membranous glomerulonephritis was diagnosed (Ehrenreich and Churg Stage I) and the patient was treated with steroid boluses (methylprednisolone 500 mg ev/die for 3 days) and Rituximab (1 gram at time 0 and after 7 days). Remission of nephrotic syndrome was obtained after 2 months.

### Case 3

A 74 year-old man was admitted to our hospital because of chronic cough, dysphonia, dysphagia, hypoacusia, fever, and weight loss. He presented chronic arterial hypertension, atrial fibrillation, and no history of allergy. At admission ESR and CRP were elevated (78 mm/H and 21.6 mg/dL, respectively; upper limits <20 and <0.5); mild anemia, neutrophilic leucocytosis, and hypergammaglobulinemia were also present. Initially, a diagnosis of cryptogenetic bronchiolitis was made on the basis of CT scan findings (pulmonary involvement with thickening of bronchial walls and a “tree-in-bud” sign) and the patient was treated with broad-spectrum antibiotics and low dose steroids. For the recurrence of fever with an increase of acute phase reactants and a progressive weakness, further examinations were performed. Autoantibody evaluation demonstrated ANA 1:80 (speckled pattern) and ANCA-MPO highly positive (84% AU, normal <18%). The rapid decline of renal function with an increase of creatinine levels from 0.81 to 2.69 mg/dL, albuminuria (100 mg/dL), micropyuria, and microhematuria strongly suggested the diagnosis of ANCA-associated vasculitis (microscopic polyangiitis, MPA). The patient was referred to our Unit in order to complete diagnostic workout and start adequate therapy.

Urinary RBP and beta2microglobulin were increased (1.9 mg/dL n.v. <0.1 and 289 mcg/L n.v. <154 mcg/L, respectively) and urinary electrophoresis showed a mixed proteinuria (glomerular and tubular) with epithelial, granular, and erythrocytic cylinders. A PET/CT scan showed a moderate 18F-fluorodeoxyglucose (18F-FDG) uptake of mediastinic, parathracheal, ilar, and axillary lymph nodes. Electroneurography and electromyography showed neurogenic damage with signs of denervation. Gadolinium-enhanced magnetic resonance imaging (MRI) of the brain disclosed a mastoiditis without other significant lesions.

Interestingly, serum IgG4 level was 529 mg/dL (normal <135 mg/dL) with an IgG4/IgG ratio of 27% and circulating plasmablasts (CD19+ CD20– CD27+ CD38^bright^ cells) were slightly increased (1,402 cells/mL; normal <635 cells/mL). We then revised the histology of a “benign lesion” of the gallbladder removed 2 years before, that was 9 cm long and induced a duodenal stenosis and a cystocolic fistula. Histologically, lymphoplasmacytic infiltrate rich of eosinophils with areas of fibrosis was detected. Immunohistochemistry for IgG4 showed abundant IgG4-positive plasma cells (IgG4+/IgG+ plasma cells ratio > 40%, 45 IgG4 ± plasmacells/HPF), consistent with IgG4-RD.

**Figure 3:**
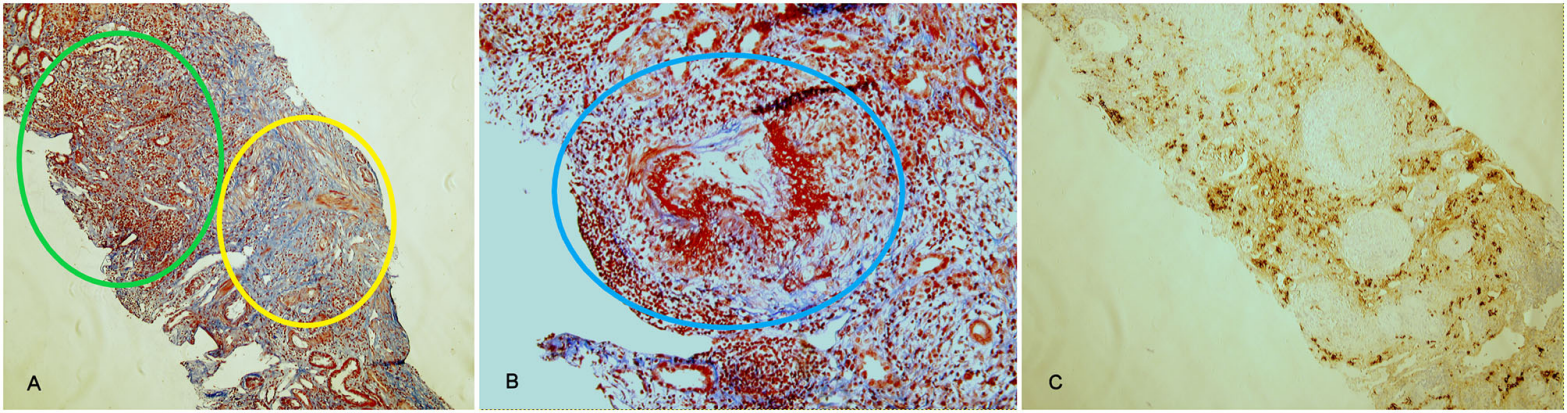
Histological findings in AAV-IgG4-RD overlap (case 3). Acute diffuse tubulointerstitial infiltrate (green circle) associated with fibrotic involvement (yellow circle) and with destruction of renal structures, highlighted by the Masson trichrome stain (**A**, 10x). Necrotizing arteritis (blue circle) (**B**, 20x). CD138 staining, highlighting plasma cells in the infiltrate (**C**, 10x). Lesions in **(A)** are suggestive of IgG4-RD, lesions in **(B)** are typical of AAV.

Finally, a renal biopsy was performed. Examination of renal parenchyma demonstrated an intense lymphomonocytic infiltrate in interstitial space, with IgG4+ plasmacells and eosinophils. In two glomeruli there was extracapillary proliferation that induced a complete rupture of Bowman capsulae. In 10% of tubuli, atrophy was detected, and 2 out of 9 glomeruli presented sclero-ialinosis. Some tubular segment, however, demonstrated signs of tubulitis with focal neutrophils in tubular epithelium and fibrinoid necrosis in medium-large vessels (Figure 3). Thus, a diagnosis of AAV-IgG4RD overlap was made. The patient underwent therapy with iv prednisone (500 mg/day for 3 days) followed by iv Cyclophosphamide (1 g/month, 7 g total dose). Six months later, creatinine level was 1.4 mg/dL; inflammatory markers (ESR 14 mm/Hg) and serum IgG4 (104 mg/dL) were normal, while ANCA-MPO antibodies, still present, were reduced in titer. Urinalysis showed absence of white and red cells and proteinuria was 250 mg/24 h.

### Case 4

A 70 years old man with multiple comorbidities (COPD, arterial hypertension, atherosclerosis with carotid, and iliac stenosis) and a previous pulmonary lobectomy with lymphadenectomy for a giant cell carcinoma, performed a total body CT scan during his oncologic follow-up. A nodular lesion (19 mm) was detected between pancreas and duodenum with contrast enhancement, and a focal pyeloureteral hypercaptation compatible with urothelial carcinoma. An abdominal MRI demonstrated 2 pancreatic lesions of the pancreas head, suspicious for malignancy. Due to the oncologic history, the patient underwent a Whipple's pancreaticoduodenectomy and left nephroureterectomy. Surgical resection was complicated by sepsis with pneumonia and a pancreatic fistula with ascites, positive for Pseudomonas Aeruginosa, that subsequently underwent surgical treatment. He then developed portal thrombosis, heparine-induced thrombocytopenia and, after 2 months, chilotorax, intraepathic aerobily, and abdominal abscess.

The histologic analysis of surgical specimens demonstrated a low-grade urothelial carcinoma, while pancreatic parenchyma showed fibrosis areas with lymphoplasmocytic infiltrates, periduttal infiltration, follicular-like aggregates, venulitis, acinar atrophy. Immunohistochemistry for IgG4 demonstrated >10 IgG4+ plasmacells/HPF and IgG4/IgG ratio >40%.

Given the aforementioned histological findings, the patient was admitted to our inpatient clinic with the suspect of IgG4-RD. Clinical and laboratory signs of sepsis were present and broad-spectrum antibiotic treatment was started, with slow improvement. During the recovery, serum IgG4 were increased (225 mg/dL, n.v. 49–66, IgG4/IgG ratio 17.78%). Interestingly, daily proteinuria was low (<100 mg/24 h) but urinary electrophoresis showed a tubular proteinuria with a small increase in urinary RBP (0.3 mg/dL, nv 0.1 mg/dL). Histological re-examination of resected kidney revealed thyroidisation of tubules, sclerotic glomeruli, severe, fibrosis of arterial walls, and interstitial lymphocytic infiltrate. An immunostaining for IgG4 showed >10 plasma cells IgG4+/HPF and a IgG4/IgG ratio >40%. Due to the frailty of the patient, a steroid therapy regimen was started in association with antibiotics. After surgical drainage of abdominal abscess, the patient's conditions improved and no relapse of IgG4-RD in any localization was observed in follow up.

## Discussion

### Tubulointerstitial Involvement

TIN is the most frequent renal manifestation of IgG4-RD and is diagnosed according to the criteria developed by the Mayo Clinic group and the Japanese Society of Nephrology (10, 11). At variance with other forms of TIN (drug-induced or associated with systemic autoimmune disorders as Sjogren's syndrome), the involvement of renal parenchyma is not diffuse but rather characterized by focal lesions surrounded by normal tissue.

Histologically, the hallmark is a diffuse or multifocal lymphocytic infiltrate with a predominance of IgG4+ plasma cells, an IgG4/IgG-positive plasma cell ratio >40% and >10 IgG4-positive plasma cell per high-power field (12). However, as the disease is zonal, the absence of of IgG4-rich plasma cell infiltrate in a kidney biopsy cannot exclude IgG4-RD. Eosinophils are frequently detected, while obliterative phlebitis is rarely seen (9). The infiltrate may affect well-demarcated areas of renal parenchyma, or rather extend into kidney capsule (13). Tubulitis is also present, with mononuclear cells, plasma cells and eosinophils (13), in the absence of cell necrosis. The fibrotic interstitium shows the typical storiform fibrosis but sometimes a different pattern can be detected, characterized by irregular fibers surrounding inflammatory cell clusters, defined “bird's eye fibrosis” (8, 14). According to the degree of interstitial inflammation and fibrosis, 3 different patterns of IgG4-TIN were described: acute TIN with minimal interstitial fibrosis; chronic TIN with expansile interstitial fibrosis; and advanced sclerosing pattern, with fewer inflammatory cells (15).

By immunofluorescence, granular deposits of IgG and C3 can be detected on tubular basal membrane; C1q can occasionally be present and electron microscopy shows electron dense deposits in the membrane.

Serologically, patients affected by TIN have no distinguishing feature except for hypocomplementemia, present in 60% of the patients, with decreased levels of C3 and/or C4, and higher serum levels of IgG and IgG4. CRP is rarely elevated, at variance with other forms of TIN (16). Acute or chronic renal insufficiency can be present, associated with a variable degree of hematuria and proteinuria. In contrast with drug-related TIN, urinary WBCs or casts are not a constant finding (16).

Radiographic lesions of IgG4-RD TIN are best visualized by contrast-enhanced computer tomography scan. Multiple low-density lesions with mild enhancement in delayed phase on enhanced CT are the most common radiological findings (11, 16). By MRI, renal lesions appear iso- or hypointense in comparison with normal renal parenchyma on T1-weighted images, hypointense on T2-weighted images.

PET/CT, useful to evaluate retroperitoneal involvement, is not advised to study renal disease, because of the interference due to kidney excretion of radio-labeled drug.

Multiple and bilateral lesions are usually detected, predominantly involving the renal cortex. Four patterns of parenchymal lesions are described: small, sub-centimetrical peripheral cortical nodules, round, or wedge- shaped lesions, diffuse patchy involvement, or (more rarely) a solitary mass (17). Nephromegaly (>14.5 cm), sometime reversible after therapy, and renal pelvis involvement were also reported (10). Lymphoma, vasculitis, pyelonephritis, and metastatic cancer should be considered in the radiographic differential diagnosis of renal parenchymal lesions.

### Glomerular Involvement

Different types of glomerular involvement are described in IgG4-RD, but membranous glomerulonephritis, occurring in roughly 7% of the patients, is the most frequent. In fact, only anecdotical reports of IgA nephropathy, endocapillary proliferative glomerulonephritis and membranoproliferative glomerulonephritis have been published (18–22).

In most cases, glomerular involvement coexists with TIN but occasionally it occurs in the absence of tubular involvement. Proteinuria in the nephrotic range is the usual manifestation of the disease. IgG and C3 deposits are detected in glomeruli by immunofluorescence and abundant electron dense deposits are shown in subepithelial space by electron microscopy (23). In a low percentage of cases, biopsy samples exhibit also mesangial or subendothelial deposits (10). The specificity of autoantibodies involved in the formation of these deposits is not known, but it is of interest the detection of antibodies against carbonic anhydrase II, a podocyte antigen, in IgG4-RD membranous nephritis (24).

Differential diagnosis from primary membranous nephropathy is based on the simultaneous presence of TIN, on the detection of other localizations of the disease (the kidney is very rarely the only organ affected), on the absence of antibodies specific for phospholipase A2 receptor. However, it is important to take into account that this autoantibody, marker of primary membranous nephropathy, is present in only 70% of the patients (21). The recent ACR/EULAR IgG4-RD classification criteria consider anti-PLA2R as an exclusion criterion for the diagnosis of IgG4-RD (6).

Recently, the coexistence of IgG4-RD and primary membranous nephropathy has been described in a patient positive for antibodies to the phospholipase A2 receptor (25).

### Retroperitoneal Fibrosis

Idiopathic retroperitoneal fibrosis (IRF) is a rare fibro-inflammatory disease of unknown etiology characterized by periaortic and peri-iliac fibrosis. It was described for the first time in 1905 and classified in 1948 by Ormond (26). It was estimated that IRF is associated to IgG4-RD in more than half of the cases, sometimes representing the only manifestation of the disease (27, 28).

The expansion of fibrosis can entrap any of the structures in the retroperitoneum, especially ureters, inducing obstructive nephropathy. Clinical features are often non-specific: lower back and flank dull pain in over 90% of patients, anorexia, weight loss, fatigue. Legs edema associated to deep venous thrombosis can be detected when vena cava obstruction is present.

Recently, it has been reported that IgG4-RD RF is characterized by lower serum IgG4 levels and a lower IgG4-RD Responder Index at disease onset if compared with other clinical phenotypes of IgG4-RD (29, 30). Histologically, the counterpart of the RF phenotype is a lower number of plasma cells in comparison with other involved organs. However, this finding could simply depend on samples obtained in more advanced stages of the disease, when the fibrotic component is prevalent.

To evaluate retroperitoneal lesions, enhanced CT scan, MRI, or PET/CT are the preferred tools; hydronephrosis can easily be detected by ultrasound. Other localizations of the disease can be more easily biopsied and can suggest the correct diagnosis.

When acute ureteral obstruction occurs, ureteral stent, percutaneous nephrostomy, or ureteral stricture surgery are necessary to overcome hydronephrosis and progression to end-stage renal disease. However, many patients can still suffer from chronic kidney disease, because of late or ineffective medical treatment (31).

### Overlap With ANCA-Associated Vasculitis (AAV)

The coexistence of IgG4-RD and AAV has been reported and a biopsy-proven overlap was described in several cases.

Chang et al. (32, 33) described eight cases out of a cohort of 43 GPA patients characterized by a high number of IgG4+ plasma cells infiltrating the kidneys, but they considered these cases as diagnostic pitfalls rather than AAV-IgG4-RD overlap. Thus, the exact incidence of AAV-IgG4-RD overlap is presently unknown, since many cases were diagnosed previously as AAV. Danlos (34) in a multicentric European retrospective study identified 10 biopsy proven overlap, but the total number of AAV patients evaluated in the study is not reported. A recent retrospective Mexican study on association between AAV and other autoimmune disease identifies only one patient out of 147 with AAV-IgG4-RD overlap (35), while another study with a more limited number of patients failed to identify any overlap with IgG4-RD (36). Thus, the incidence of AAV-IgG4-RD overlap might be estimated in 1:150 AAV, or lower.

In AAV-IgG4-RD overlap, kidney lesions attributable to both disorders can coexist and cooperate to induce anatomical and functional damage. Under this respect, the case we describe in this report is of particular relevance, because renal histology shows a glomerulonephritis compatible with AAV, with rupture of Bowman capsulae and fibrinoid necrosis, together with a tubulointerstitial involvement and IgG4+plasmacells, more typical of IgG4-RD. The coexistence of TIN with eosinophilic infiltrate has been previously described in AAV, before the identification of IgG4-RD as distinct nosological entity (37). In the light of more recent findings, some of these cases might be more correctly diagnosed as AAV-IgG4-RD overlap.

ANCA are critical for the differential diagnosis between AAV and IgG4-RD, but low titer ANCA are present in several autoimmune disorders including IgG4-RD. Thus, the detection of IgG4 ANCA has been taken into account as possible tool for diagnosis.

ANCA positivity is one of the exclusion criteria proposed for the diagnosis of IgG4-RD in ACR/EULAR IgG4-RD classification criteria (6).

However, IgG1 and IgG4 ANCA are reported by Della-Torre et al. (38) in a case of AAV-IgG4-RD overlap, and also by Su et al. (39). Similarly, Abbass et al. (40) claim that in their clinical case ANCA were predominantly IgG4. However, data on IgG2 and IgG3 ANCA in these patients are not available.

Comparing the subclass distribution of ANCA MPO in AAV and IgG4-RD, we found that ANCA MPO of any subclass, and not only IgG4, can be detected in IgG4-RD sera. However, the titer of these antibodies is very low in comparison with AAV; moreover, antibody titer and the number of ANCA subclasses are not correlated to clinical activity, at variance with the results obtained in AAV. Thus, ANCA titer seems more relevant than subclass distribution for differential diagnosis and the detection of ANCA-MPO at high titer, without analyzing ANCA subclasses, is sufficient to corroborate serologically the clinical suspect of an AAV-IgG4-RD overlap (41).

### Treatment and Follow-Up

Due to the rarity of the disease, no randomized trial specifically addressing IgG4-RKD has been conducted so far. Only single case reports have been published, and the renal involvement in IgG4-RD is treated according to the therapeutic approach used for the other localizations of the disease. Since the kidney is a critical organ, early therapy is recommended to prevent long-term fibrotic damage.

First-line treatment is still represented by steroids, according to a 2015 consensus statement from an international expert panel on the treatment of IgG4-RD (42). Patients with early diagnosed IgG4-RD TIN present higher values of eGFR than patients with a late diagnosis of renal involvement, probably because of a prompt glucocorticoid therapy (43). Patients with eGFR <60 ml/min/1.73 m^2^, as well as advanced histological stage, still present an improvement after steroid treatment, albeit limited (43–45), but are at major risk to develop renal cortical atrophy (46). Repeated renal biopsy in IgG4-RD TIN patients during steroid treatment shows in fact progressive fibrosis of the interstitium, despite a reduction of inflammatory infiltrate (47, 48). Despite a good response to steroids in almost all IgG4-RD patients, the relapse rate is high at tapering, and nearly 40% of patients fail to achieve remission after 1 year (49, 50). These data suggest that different strategies should be used to properly control the disease.

Many DMARDs have been employed in IgG4-RD, such as methotrexate, azathioprine, tacrolimus, but their efficacy in maintaining a relapse-free condition, with or without a low-dose steroid, has not yet been verified. Up to now, only two trials were conducted on classical DMARDs, Mycophenolate Mofetil (MMF) and oral Cyclophosphamide (CFX) (51, 52). Both drugs are effective on relapse rate in comparison with steroid-only regimen: after 1 year of therapy, relapses are observed in 20% of patients treated with MMF and in 12% treated with oral CFX, respectively.

B cell-targeted therapy with Rituximab (RTX), a monoclonal antibody anti CD-20, leads to an excellent response, with a remarkable reduction of mass lesions and a variable regression of fibrosis even in a steroid-free regimen (53). RTX administration reduces serum IgG4 level and circulating plasmablasts (54). In a case report, RTX alone substantially improved renal function in a 58-year-old man with IgG4-RD TIN (55). RTX, that depletes mature B cells but spares plasma cells, has a better safety profile than CFX, less toxicity and fewer infectious events. Albeit widely used, a specific evaluation of rituximab efficacy in renal involvement is still lacking. It has to be taken into account that RTX modulation of disease activity occurs 4–8 weeks after the infusion. Thus, Quattrocchio et al. proposed a treatment protocol for IgG4-TIN that includes steroid boluses, 2 pulses of iv CFX and 4 weekly doses of RXT; this protocol showed a sustained efficacy, both clinically and histologically, over a 4-year follow-up (56, 57).

In contrast, there is no clear demonstration that an exclusive steroid regimen is an effective approach for IgG4-membranous nephropathy (58). Patients are managed as primary membranous nephropathy with Rituximab, cyclophosphamide, or cyclosporine.

Notably, the disease can recur in the transplanted organ, as reported by Chibbar et al. Despite therapy with prednisone (5 mg daily), tacrolimus 6–8 mcg/L, and mycophenolate mofetil 2 g daily, a 25 year old patient had relapse of IgG4-TIN 5 years after renal transplant, concomitant with chronic active antibody mediated rejection (59).

IgG4-RD RF benefits of the same treatments already described. Notably, recent reports of MMF and RTX treatment in RF show efficacy whether or not it is a manifestation of IgG4-RD (60, 61). Some conditions, such as smoking habit, AKI at diagnosis, ANA positivity and lumbar pain, are associated to relapse, and all these factors represent a negative prognostic combination for renal outcome (62).

## Conclusions

Renal involvement in IgG4-RD is a dangerous condition that can lead to a chronic organ damage, potentially life-threatening. Multiple reports suggest the need of an aggressive therapy in order to avoid a fibrotic remodeling and a functional damage.

Describing four clinical cases, we moved from sunlight to twilight, going from the classical pattern of renal involvement of case 1 and 2 to the more complex picture of case 3 and finally to the subclinical involvement of case 4.

These four patients enlight the heterogeneity of clinical manifestations as distinguishing feature of IgG4-RD also when affecting the kidney.

Thus, a careful periodic assessment of kidney function with evaluation of filtration rate and urinary sediment, in association with adequate imaging, is mandatory for each IgG4-RD patient. Presently, urinary markers that allow an early diagnosis and follow up of renal involvement in IgG4-RD are not available.

More investigations are needed to define biomarkers (urinary cells, urinary cytokines/mediators) able to correctly profile the type and extent of renal involvement in IgG4-RD.

## Data Availability Statement

The raw data supporting the conclusions of this article will be made available by the authors, without undue reservation.

## Ethics Statement

Ethical review and approval was not required for the study on human participants in accordance with the local legislation and institutional requirements. The patients/participants provided their written informed consent to participate in this study.

## Author Contributions

RC, DG, and PM conceived the work and wrote the paper. DG, DM, and ME were in charge of nephrological evaluation of the patients. DG performed renal biopsies. AB performed biopsies examination. CC and FP performed immunohistochemical and cytofluorimetric evaluations. RC, IP, and AT were in charge of the patients and collected clinical data. All authors contributed to the article and approved the submitted version.

## Conflict of Interest

The authors declare that the research was conducted in the absence of any commercial or financial relationships that could be construed as a potential conflict of interest.

## Supplementary Material

Supplementary Figure 1 Immunofluorescence staining of Case # 2 kidney biopsy: few igG1+ cells were observed but no IgG2+ or IgG3+ cells.
