# Supplementary figures and images for: Renal Involvement in IgG4-Related Disease: From Sunlight to Twilight
Source: Front Med (Lausanne). 2021 Mar 31;8:635706. doi: 10.3389/fmed.2021.635706 (PMC8044528; doi:10.3389/fmed.2021.635706)

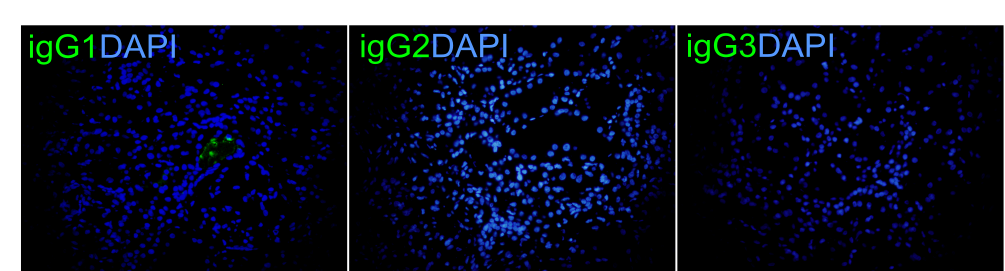

Supplement: Supplementary Figure 1 — Immunofluorescence staining of Case # 2 kidney biopsy: few igG1+ cells were observed but no IgG2+ or IgG3+ cells. [file Image_1.tiff]
